# G protein β_4_ as a structural determinant of enhanced nucleotide exchange in the A_2A_AR-Gs complex

**DOI:** 10.21203/rs.3.rs-3814988/v1

**Published:** 2024-01-23

**Authors:** William E. McIntire, Michael D. Purdy, Susan A. Leonhardt, Iga Kucharska, Michael A. Hanson, Sandra Poulos, James C. Garrison, Joel Linden, Mark Yeager

**Affiliations:** 1 The Phillip and Patricia Frost Institute for Chemistry and Molecular Science, University of Miami, Coral Gables, Florida 33146.; 2 Department of Molecular Physiology and Biological Physics, University of Virginia School of Medicine, Charlottesville, VA 22908 USA.; 3 Molecular Electron Microscopy Core, University of Virginia School of Medicine, Charlottesville, Virginia 22908, USA.; 4 Department of Pharmacology, University of Virginia Health System, Charlottesville, VA 22903 Virginia 22908, USA.

## Abstract

Adenosine A_2A_ receptors (A_2A_AR) evoke pleiotropic intracellular signaling events via activation of the stimulatory heterotrimeric G protein, Gs. Here, we used cryoEM to solve the agonist-bound structure of A_2A_AR in a complex with full-length Gs α and Gβ_4_γ_2_ (A_2A_AR-Gs α:β_4_γ_2_). The orthosteric binding site of A_2A_AR-Gs α:β_4_γ_2_ was similar to other structures of agonist-bound A_2A_AR, with or without Gs. Unexpectedly, the solvent accessible surface area within the interior of the complex was substantially larger for the complex with Gβ_4_ versus the closest analog, A_2A_AR-miniGs α:β_1_γ_2_. Consequently, there are fewer interactions between the switch II in Gs α and the Gβ_4_ torus. In reconstitution experiments Gβ_4_γ_2_ displayed a ten-fold higher efficiency over Gβ_1_γ_2_ in catalyzing A_2A_AR dependent GTPγS binding to Gs α. We propose that the less constrained switch II in A_2A_AR-Gs α:β_4_γ_2_ accounts for this increased efficiency. These results suggest that Gβ_4_ functions as a positive allosteric enhancer versus Gβ_1_.

## Introduction

Heterotrimeric G proteins, composed of a Gα subunit and Gβγ dimer, interact with hundreds of different cell surface GPCRs, transducing extracellular stimuli such as light, odorants and hormones to intracellular effectors ^1^. The Gs-coupled A_2A_ adenosine receptor (A_2A_AR) has been an important therapeutic target for ameliorating the effects of inflammation ^2^, cancer ^3^, cardiovascular^4^ and neurological disease ^5^.

Upon binding agonist, the A_2A_AR, aided by Gβγ, catalyzes the exchange of GDP for GTP on Gs α, eliciting activation. The GTP bound Gs α has a lower affinity for Gβγ, resulting in the dissociation of the Gs heterotrimer from A_2A_AR, thereby liberating Gs α and Gβγ ^6^, which can regulate effectors such as adenylyl cyclase to increase intracellular cAMP. GTP bound Gs α is deactivated by an intrinsic GTPase activity that converts GTP to GDP, a process that can be accelerated by Regulators of G protein Signaling (RGS) proteins ^7^. The GDP bound Gs α has a higher affinity for Gβγ and can bind sites on Gβ that overlap effector binding sites, which inhibits signaling by Gβγ. The reassembled Gs heterotrimer then binds the plasma membrane to repeat the cycle with another GPCR.

**Fig. 1 |:**
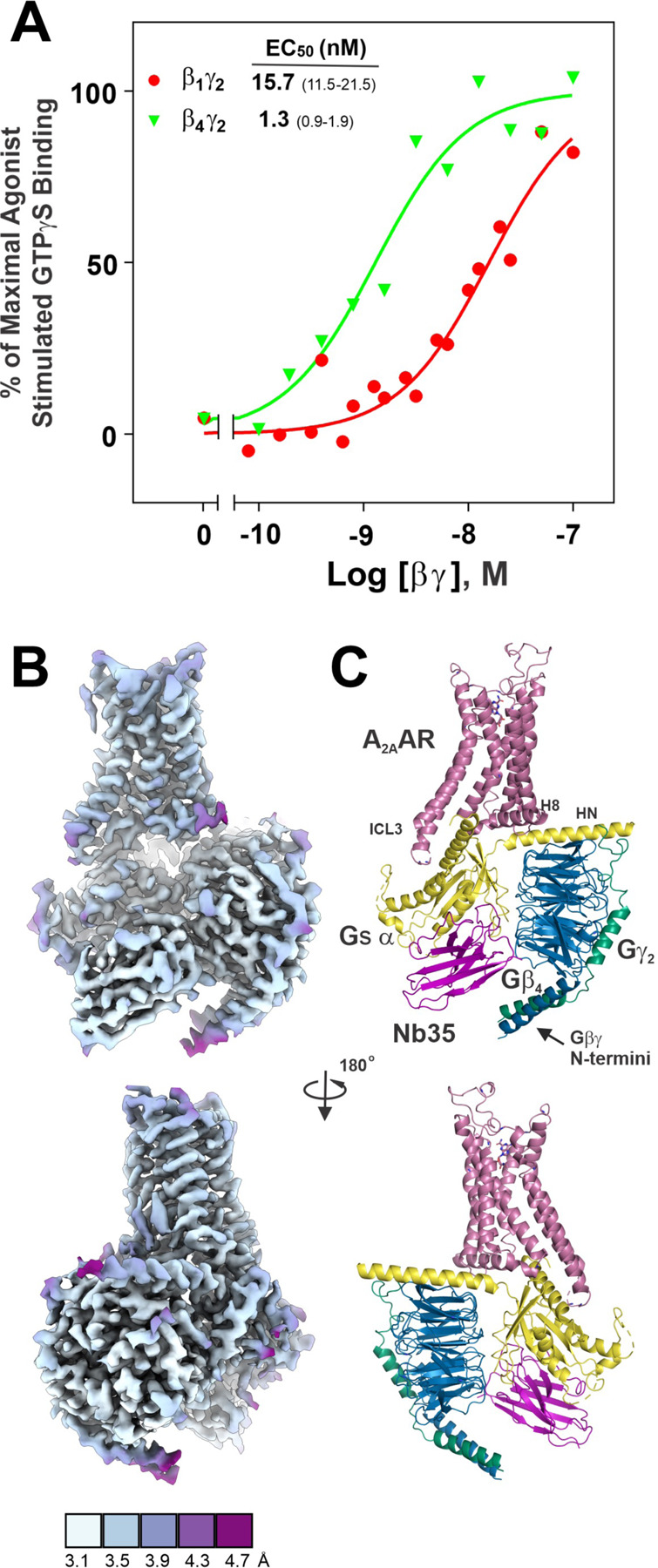
CryoEM structure of the A_2A_AR-Gs complex containing the Gβ_4_ isoform. (**A**) Published data from McIntire et al. ^9^ demonstrating ten-fold higher efficiency of Gβ_4_γ_2_ over Gβ_1_γ_2_ in catalyzing A_2A_AR dependent GTPγS binding to Gs α. (**B**) Two views of the A_2A_AR-Gs complex CryoEM map colored and filtered by local resolution. (**C**) Ribbon representation of CryoEM map of the A_2A_AR-Gs complex: A_2A_AR-reddish purple, Gs α-yellow, Gβ_4_-blue, Gγ_2_-bluish green, Nb35-magenta.

The magnitude of A_2A_AR signaling can be modulated by a variety of factors. For example, stimulation of A_2A_AR by a partial agonist results in a modest rate of nucleotide exchange in Gs, whereas a full agonist produces a more robust level of nucleotide exchange ^8^. These differences in activity have been ascribed to distinct active states of the A_2A_AR-Gs complex, which have the same affinity between Gs and the A_2A_AR, but can be distinguished by structural differences in the vicinity of transmembrane helix 6 (TM6) of the A_2A_AR, as measured by Fluorine NMR ^8^. In addition, the composition of the Gβγ dimer in a Gs heterotrimer can affect the coupling efficiency at the A_2A_AR, exemplified by the ten-fold increase in efficiency of Gβ_4_γ_2_ over Gβ_1_γ_2_ at catalyzing A_2A_AR dependent GTPγS binding to purified Gs α ^9^ (Fig. 1A). Furthermore, the Gs α:β_4_γ_2_ heterotrimer can convert a much higher proportion of A_2A_AR into the high affinity state than Gs α:β_1_γ_2_^10^. However, the high ligand binding affinity of A_2A_AR was the same, whether the Gs heterotrimer contained Gβ_1_ or Gβ_4_.

One condition where modulation of A_2A_AR signaling would be biologically important is inflammation ^11^. For example, inflammatory cytokines upregulate expression of the A_2A_AR in human dermal microvascular endothelial cells ^12^; which increases A_2A_AR signaling through Gs, resulting in increased cellular cAMP, countering the effects of inflammation. However, in addition to increasing A_2A_AR expression, inflammatory cytokines also increase Gβ_4_ expression ^12^. Considering the functional attributes of Gβ_4_ with respect to the A_2A_AR, increasing the ratio of A_2A_AR in the high affinity state, and increasing the efficiency of nucleotide exchange, it is not surprising that a cell would increase expression of both A_2A_AR and Gβ_4_ to maximize signaling from A_2A_AR in response to an inflammatory stimulus.

Understanding the mechanism by which Gβγ improves receptor coupling by 10-fold is essential for complete understanding of GPCR-G protein signaling. To this end, we solved the first cryoEM structure of agonist-bound A_2A_AR in a complex with full-length Gs α and the novel Gβ_4_γ_2_ heterodimer. Comparison of the A_2A_AR-Gs α:β_4_γ_2_ complex with the previously solved A_2A_AR-miniGs α:β_1_γ_2_ complex ^13^ enabled interrogation of structural differences between Gβ_1_γ_2_ and Gβ_4_γ_2_. Surprisingly, A_2A_AR-Gs α:β_4_γ_2_ displayed significantly greater solvent accessible surface area that we propose accounts for the ten-fold increased efficiency of Gβ_4_γ_2_ over Gβ_1_γ_2_ in catalyzing A_2A_AR dependent GTPγS binding to Gs α. These results suggest that Gβ_4_ functions on the cytoplasmic surface as a positive allosteric modulator, like partial and full agonists on the extracellular surface.

## Results

### Biophysical and functional and characterization of A_2A_AR-Gs complexes

To investigate the role of Gβ_4_ in A_2A_AR-Gs signaling, we used anti-FLAG M1 affinity resin (Supplemental Fig. 1A) to form a complex between heterotrimeric Gs α:β_4_γ_2_ and A_2A_AR with a bound agonist UK-432,097 (Supplemental Fig. 1B). The eluted A_2A_AR-Gs α:β_4_γ_2_ complex was monodisperse as judged by size exclusion chromatography (Supplemental Fig. 1C) and free of contaminating proteins as visualized by SDS-PAGE (Supplemental Fig. 1D). Functional activity of the A_2A_AR-Gs α:β_4_γ_2_ complex was verified by the presence of GTPγS sensitive high affinity agonist binding, as measured by 5′-(N-Ethylcarboxamido)adenosine (NECA) displacement of bound ^3^[H] ZM-241,385 (Supplemental Fig. 1E).

### Structures of A_2A_AR and A_2A_AR-Gs α:β_4_γ_2_

In the course of our work on solving the A_2A_AR-Gs complex structure, we solved the X-ray structure of agonist-bound A_2A_AR. The A_2A_AR construct for lipidic cubic phase crystallization contained T4 lysozyme (T4L) inserted in intracellular loop 3 (ICL3), and the structure was nearly identical to the published X-ray structure of A_2A_AR with the bound agonist UK-432,097 (RMSD 0.395 Å) ^14^. As noted above, the Gβ_4_γ_2_ heterodimer was ten-fold more efficacious than the Gβ_1_γ_2_ heterodimer in catalyzing A_2A_AR dependent GTPγS binding to Gs α^9^ (Fig. 1A), which was the overarching rationale for solving an A_2A_AR-Gs structure containing Gβ_4_. For our cryoEM studies, expression of A_2A_AR in Sf9 insect cells was increased by truncating the C-tail and including an N-terminal T4L fiducial marker. Single-particle cryoEM of A_2A_AR-Gs α:β_4_γ_2_ yielded a Coulomb potential (CP) map with a resolution ranging between 3.1 and 4.7 Å with an overall, gold-standard Fourier shell correlation (FSC) resolution of 3.5 Å (Fig. 1B). Corresponding models of the A_2A_AR-Gs α:β_4_γ_2_ complex (Fig. 1C) recapitulate the general architecture of A_2A_AR in a complex with Gα (lacking the α-helical domain (so-called miniG)) and β_1_γ_2_^13^.

### The interior solvent accessible volume is larger in A_2A_AR-Gs α:Gβ_4_γ_2_ versus A_2A_AR-miniGs α:Gβ_1_γ_2_.

**Fig. 2 |:**
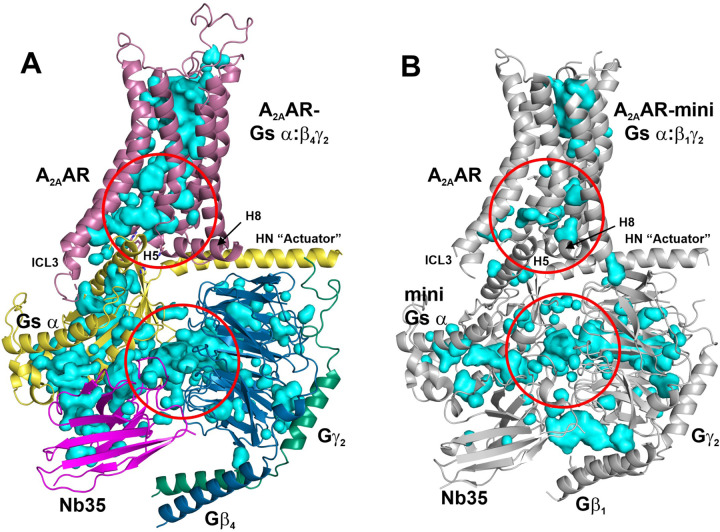
The interior solvent accessible volume is larger in A_2A_AR-Gs α:Gβ_4_γ_2_ versus A_2A_AR-miniGs α:Gβ_1_γ_2_. **(A)** Structure of A_2A_AR-Gs α:Gβ_4_γ_2_ illustrating cavities (cyan) generated in PyMOL with a cavity detection radius of 3 solvent radii and a cavity cutoff of 4 solvent radii. Upper red circle indicates the larger cavity in the area between the orthosteric binding site and the G protein binding site. Lower red circle indicates the larger cavity in the interface between Gs α and Gβ_4_. (**B**) Structure of A_2A_AR-miniGs α:Gβ_1_γ_2_ illustrating cavities as described in (**A**). Upper red circle indicates same area as described in (**A**) with smaller cavity. Lower red circle indicates the same area as described in (**A**) with smaller cavity.

**Fig. 3 |:**
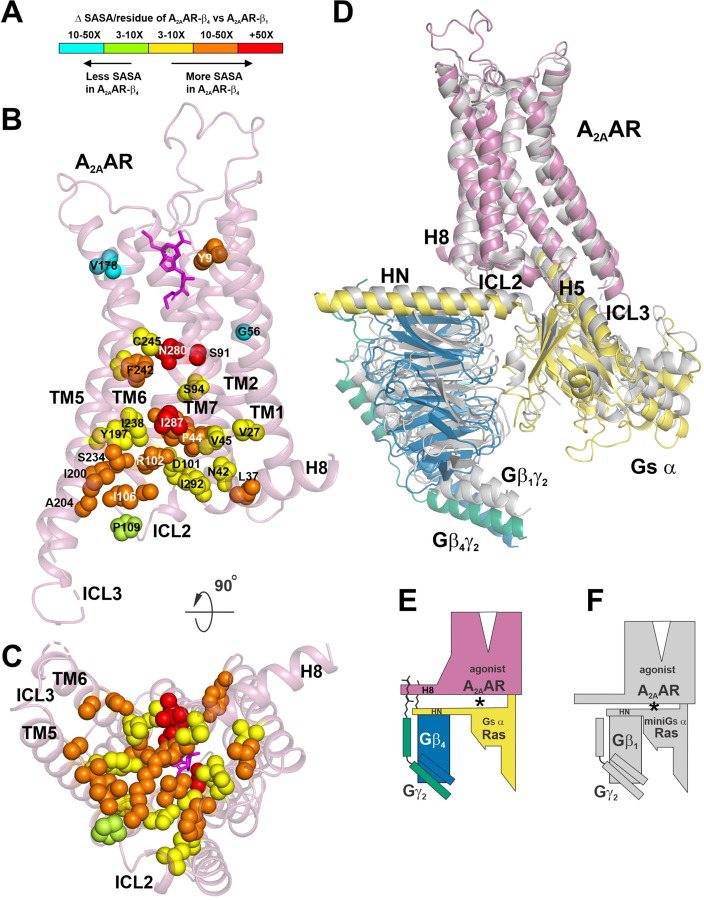
The increase in SASA at the cytosolic side of the A_2A_AR in A_2A_AR-Gs α:Gβ_4_γ_2_ is associated with an increased distanced between A_2A_AR and Gs α:Gβ_4_γ_2_. (**A**) Color scale indicating differences in SASA. (**B**) Structure of the A_2A_AR from A_2A_AR-Gs α:Gβ_4_γ_2_ highlighting residues that have significantly different solvent accessibility compared with the analogous residues in A_2A_AR from A_2A_AR-miniGs α:Gβ_1_γ_2_ (**PDB: 6GDG**). Colored spheres at the position of specific residues are used to indicate 10–50 fold (cyan) and 3–10 fold (light green) less solvent accessible surface area, or 3–10 fold (yellow), 10–50 fold (orange) and +50 fold (red) greater solvent accessible surface area in A_2A_AR from A_2A_AR-Gs α:Gβ_4_γ_2_ versus A_2A_AR-miniGs α:Gβ_1_γ_2_. (**C**) Bottom view of (**B**) rotated 90°. (**D**) Superposition of A_2A_AR-Gs α:Gβ_4_γ_2_ (colored) and A_2A_AR-miniGs α:Gβ_1_γ_2_ (grey), using the A_2A_AR for alignment. Note the additional space between A_2A_AR and Gs α:Gβ_4_γ_2_. (**E**) Cartoon rendering of A_2A_AR-Gs α:Gβ_4_γ_2_ and (**F**) A_2A_AR-miniGs α:Gβ_1_γ_2_ emphasizing increased distance between A_2A_AR and Gs α:β_4_γ_2_ (see asterisks).

Structural alignment can be very useful for examining differences in subdomains between similar proteins; however, comparison of global structural changes in A_2A_AR, or any GPCR, under different conditions by alignment is inherently challenging. Significant differences can be averaged into many small changes throughout the receptor, which can be difficult to interpret. Upon initial inspection, the A_2A_AR from the complex presented here did not appear to differ from the A_2A_AR in the A_2A_AR-miniGs α:Gβ_1_γ_2_^13^ (RMSD 1.22 Å). Not surprisingly, comparisons of the orthosteric binding site to other structures containing agonist bound A_2A_AR were also very similar, including UK-432,097 bound A_2A_AR-Gs α:Gβ_4_γ_2_, NECA bound A_2A_AR-miniGs α:Gβ_1_γ_2_^13^, NECA bound A_2A_AR-miniGs α ^15^, and UK-432,097 bound A_2A_AR ^14^. However, measurement of the solvent accessible surface area (SASA) is an orthogonal tool that can also be used to interrogate subtle structural differences between the same protein under different conditions. Using this technique, the solvent accessible volume below the orthosteric binding site and above the G protein binding site was larger in A_2A_AR-Gs α:β_4_γ_2_ versus A_2A_AR-miniGs α:β_1_γ_2_ (Fig. 2A, B, upper red circles), which was quantified in Fig. 3B, C. In particular, amino acid residues at the lower half of the receptor that interact with the G protein have increased SASA in A_2A_AR-Gs α:β_4_γ_2_ versus A_2A_AR-miniGs α:β_1_γ_2_. (Fig. 3D–F). Note also that alignment of receptors emphasizes the downward shift of Gs in A_2A_AR-Gs α:β_4_γ_2_ versus A_2A_AR-miniGs α:β_1_γ_2_ (Fig. 3D), which increases the solvent accessible volume (Fig. 2A). In addition, the cavity space is increased between Gs α and Gβ_4_ versus miniGs α and Gβ_1_ (Fig. 2A, B, lower red circles).

### The increased SASA in A_2A_AR-Gs α:β_4_γ_2_ elicits a proline switch in TM7.

**Fig. 4 |:**
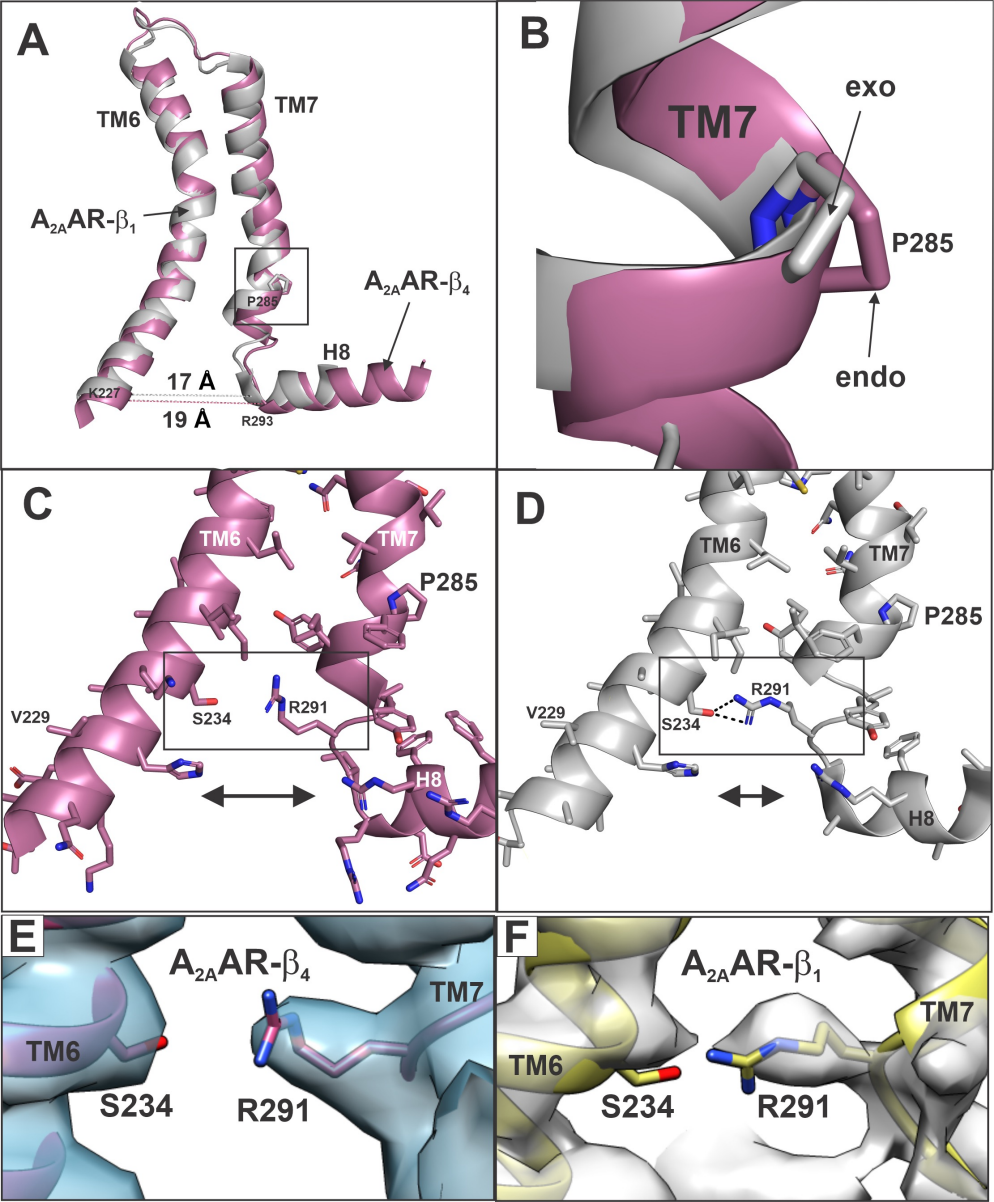
The proline 285 switch in A_2A_AR TM7 is associated with enlargement of the Gs α binding site in A_2A_AR-Gs α:Gβ_4_γ_2_. **(A)** Superposition of TM6, TM7 and H8 of A_2A_AR from A_2A_AR-Gs α:Gβ_4_γ_2_ (reddish purple) versus A_2A_AR-miniGs α:Gβ_1_γ_2_ (grey) using TM6 for alignment. Measurements (dashed lines) are from α-carbons in K227 (TM6) to R293 (H8). (**B**) Closeup of (**A**) showing the exo conformation of P285 (TM7) in A_2A_AR from A_2A_AR-miniGs α:Gβ_1_γ_2_ and the endo conformation of P285 (TM7) in A_2A_AR from A_2A_AR-Gs α:Gβ_4_γ_2_. (**C**) TM6, TM7 and H8 of A_2A_AR from A_2A_AR-Gs α:Gβ_4_γ_2_ with side chains; note the distance (>4Å) between S234 (TM6) and R291 (TM7) preclude bond formation (see arrow). (**D**) TM6, TM7 and H8 of A_2A_AR from A_2A_AR-miniGs α:Gβ_1_γ_2_ with side chains, illustrating the hydrogen bonds (black dashed lines) between S234 (TM6) and R291 (TM7) determined using the PyMOL plugin, show contacts (see arrow). (**E**) CryoEM map of boxed area from (**C**). (**F**) CryoEM map of boxed area from (**D**).

Many of the receptor residues that displayed greater SASA in A_2A_AR-Gs α:β_4_γ_2_ were localized in TM6 and TM7 (Fig. 3B, C). Closer examination revealed that TM6 of A_2A_AR of A_2A_AR-Gs α:β_4_γ_2_ aligns very well with TM6 of A_2A_AR-miniGs α:β_1_γ_2_ (Fig. 4A). However, this alignment emphasizes differences in the TM7 backbones. P285^7.50^ (superscript refers to Ballesteros-Weinstein numbering system ^16^) of the NPXXY motif resides at the lower third of TM7, which is an important conformational switch in GPCR activation ^17^. The proline in the NPXXY motif induces a kink in TM7 (Fig. 4A) ^18^. Importantly, P285^7.50^ in A_2A_AR of A_2A_AR-Gs α:β_4_γ_2_ is in the endo conformation, whereas the analogous proline in A_2A_AR-miniGs α:β_1_γ_2_ is in the exo conformation (Fig. 4B). This switch is triggered by the increased solvent accessible volume in A_2A_AR-Gs α:β_4_γ_2_, beginning at the proline 285 kink (Fig. 4A). A consequence of the increased solvent accessible volume is a loss of the hydrogen bond between S234^6.36^ in TM6 and R291^7.56^ in TM7 in A_2A_AR-Gs α:β_4_γ_2_ (Fig. 4C), whereas this hydrogen bond is retained in A_2A_AR-miniGs α:β_1_γ_2_ (Fig. 4D). This interpretation is supported by the cryoEM maps (Fig. 4E, F).

### Expansion of the Gs α-Gβ_4_ interface elicits an extension in the N-terminal helix (HN) of Gs α, which functions as an “actuator” to enlarge the cavity of the complex.

**Fig. 5 |:**
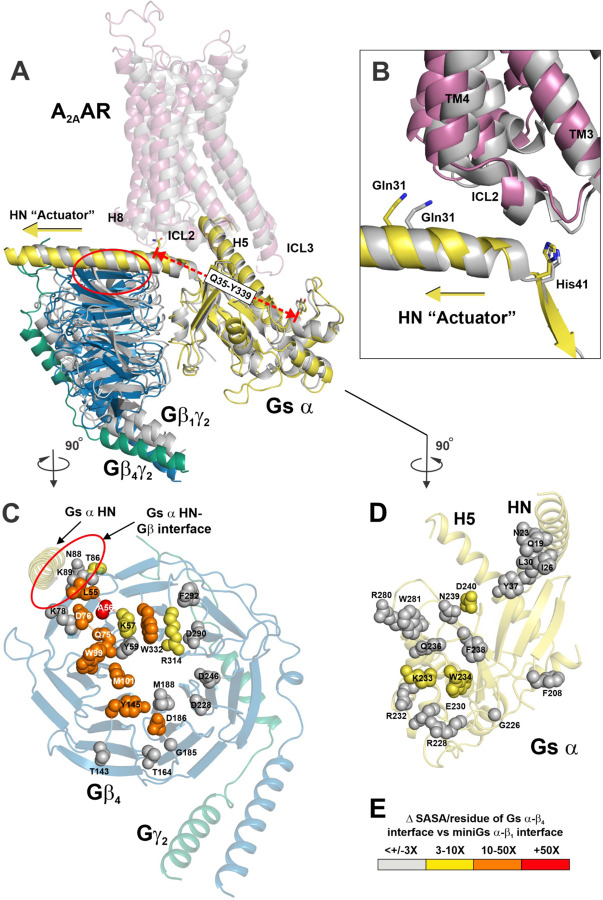
The N-terminal helix (HN) of Gs α serves as an “actuator” linking ICL2 and the Gβ torus, facilitating Gα-Gβ interface expansion in A_2A_AR-Gs α:β_4_γ_2_ versus A_2A_AR-miniGs α:β_1_γ_2_. (**A**) Superposition of A_2A_AR-Gs α:Gβ_4_γ_2_ (colored) and A_2A_AR-miniGs α:Gβ_1_γ_2_ (grey), using the the Ras domain of Gs α for alignment. Red dashed line indicates measurement between α carbons of Q35^G.HN.52^ in HN and Y339^G.H4.08^ of H4 of Gs α. Red oval indicates interface between Gβ torus and Gs α N-terminal “actuator” helix. Note the additional space between Gs α and Gβ_4_γ_2_ and the extension of the N-terminal helix (HN) of Gs α, which appears to act as an actuator linking ICL2 with the Gβ torus (See yellow arrow). (**B**) Closeup of the ICL2-HN interaction from (**A**), emphasizing the HN extension (yellow arrow) that occurs between Gln31 and His41 in Gs α from A_2A_AR-Gs α:Gβ_4_γ_2_. (**C**) Gβ_4_γ_2_ from A_2A_AR-Gs α:Gβ_4_γ_2_ in (**A**) rotated 90° clockwise. (**D**) Gs α from A_2A_AR-Gs α:Gβ_4_γ_2_ in (**A**) rotated 90° counterclockwise. Note that Gs α N-terminal helix is shown with the Gβ_4_γ_2_ (left in **C**) to emphasize the interface between Gs α HN and the Gβ torus (red oval). Gs α-Gβ contacting residues are shown as spheres that have 3–10 fold (yellow), 10–50 fold (orange) or >50 fold (red) more solvent accessible surface area in A_2A_AR-Gs α:Gβ_4_γ_2_ than the analogous residues in A_2A_AR-miniGs α:β_1_γ_2._ Gs α-Gβ contacting residues (< 4Å) that had less than +/− 3-fold difference in SASA are indicated by grey spheres. (**E**) Color scale indicating differences in SASA.

**Fig. 6 |:**
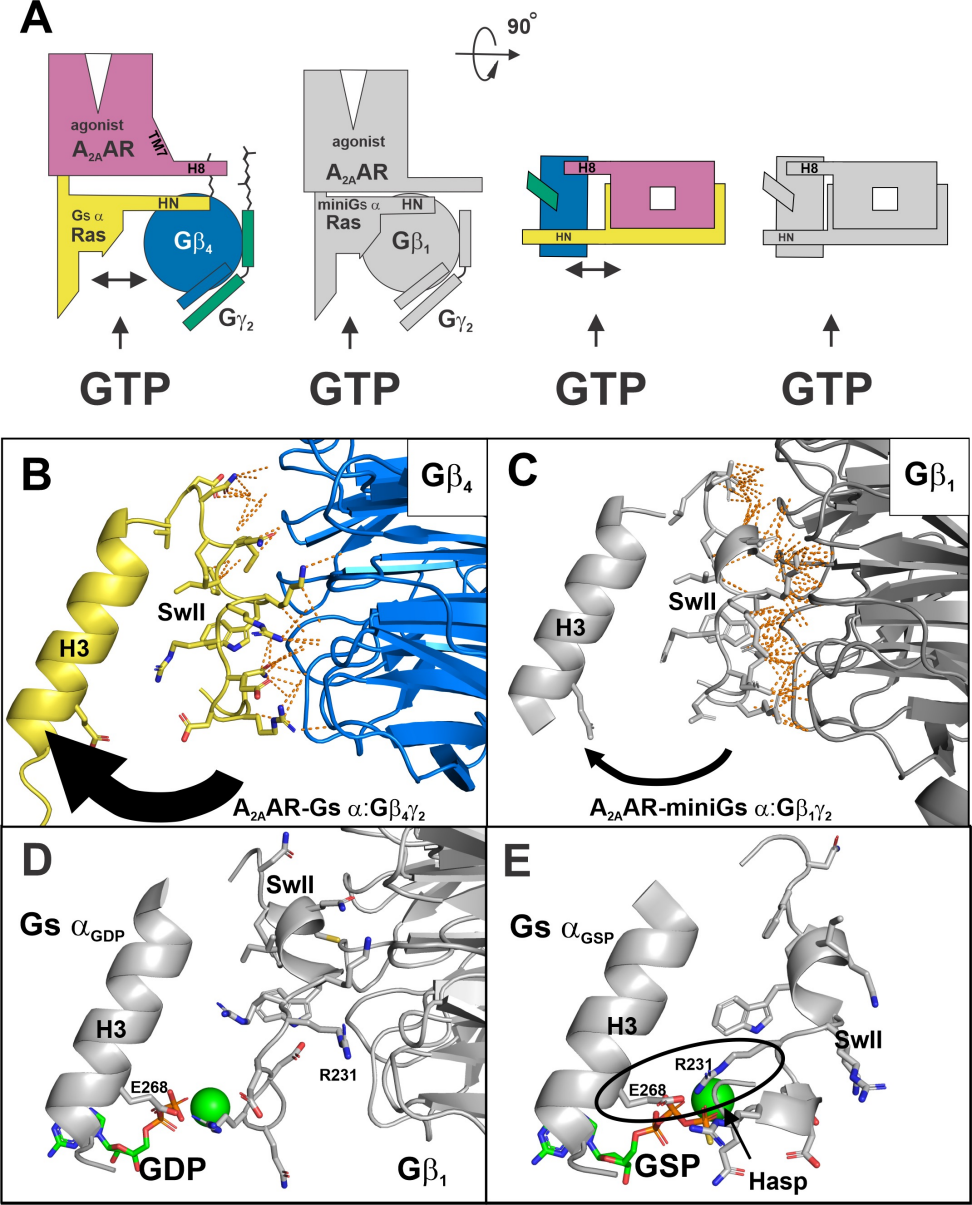
Movement of the Gs α switch II is less constrained in A_2A_AR-Gs α:Gβ_4_γ_2_ versus A_2A_AR-miniGs α:Gβ_1_γ_2_. **(A)** Cartoon rendering of A_2A_AR-Gs α:Gβ_4_γ_2_ and A_2A_AR-miniGs α:Gβ_1_γ_2_ emphasizing differences in shape of A_2A_AR and proximity of Gs α and Gβ; top views of the complexes are shown at right. (**B**) Ribbon model of A_2A_AR-Gs α:Gβ_4_γ_2_ highlighting H3 of Gs α (yellow) and the interface of Switch II of Gs α (yellow) and the torus of Gβ_4_; the remainder of Gs α is omitted. Contacts <4 Å between switch II and Gβ_4_ were generated using PyMOL and shown as dashed orange lines. (**C**) Comparison view of the ribbon model of A_2A_AR-miniGs α:Gβ_1_γ_2_ (**PDB: 6GDG**) highlighting H3 and Switch II of and the Switch II-Gβ_1_ Interface. Contacts <4 Å between switch II and Gβ_1_ were generated as described in (**B**). (**D**) View of H3 and Switch II of GDP bound Gs α:β_1_γ_2_ heterotrimer (**PDB: 6EG8**) and the interface between Switch II and Gβ_1_. (**E**) View of H3 and Switch II of GSP bound Gs α (**PDB: 1AZT**). Note the conformational change in Switch II, especially R231, which contacts Gβ in the GDP bound state (**D**) but forms a hasp with E268 of H3 in the activated GSP bound state (**E**).

To highlight a prominent left lateral shift of Gs α HN in A_2A_AR-Gs α:β_4_γ_2_, (gold) vs A_2A_AR-miniGs α:β_1_γ_2_ (grey), we aligned the complexes using the Gs α Ras domains (Fig. 5A). This lateral shift of HN is associated with an increase in the length of the Gs α subunit in A_2A_AR-Gs α:β_4_γ_2_ along the HN-H4 axis by > 3 Å (as measured between α carbons of residues Q35^G.HN.52^ and Y339^G.H4.08^) than the analogous measurement (Q35-Y329) in miniGs α in A_2A_AR-miniGs α:β_1_γ_2_ (Fig. 5A, red dashed line). SASA analysis of the Gs α-Gβ contact surface revealed a larger area (3x to 50x) in A_2A_AR-Gs α:β_4_γ_2_ than the analogous contact surface in A_2A_AR-miniGs α:β_1_γ_2_ (Fig. 5C, D and E). With the Ras domains aligned, we note that HN in Gs α:β_4_γ_2_ is shifted left with respect to ICL2 (Fig. 5A, red circle), compared with HN in miniGs α:β_1_γ_2_. This shift occurs between Q31 and H41 at a contact site with ICL2 (Fig. 5B). Given the tight contacts between Gs α HN and the Gβ torus (Fig. 5A and C, red ovals), the left lateral extension of HN in Gs α:β_4_γ_2_ (gold arrow) results in lateral translation of the β_4_ torus compared with the β_1_ torus (Fig. 5A), thereby increasing the SASA in Gs α:β_4_γ_2_. In this way we propose that HN functions as an “actuator” to manifest this expansion. These conformational changes are depicted as cartoons in Fig. 6A.

## Discussion

Comparison of the cryoEM structures of A_2A_AR-Gs α:β_4_γ_2_ and A_2A_AR-miniGs α:β_1_γ_2_ revealed dramatic expansion of the solvent accessible volume in the complex containing Gβ_4_ versus Gβ_1_. However, we note several caveats in this comparison. The receptor in A_2A_AR-Gs α:β_4_γ_2_ was deglycosylated by treatment with PNGaseF, whereas the receptor A_2A_AR-miniGs contained a N154A mutation to prevent glycosylation. In addition, A_2A_AR-miniGs α:β_1_γ_2_ is so named because it lacks the α-helical domain and Switch III of Gs α and contains several mutations that are distinguished from full-length, wild-type Gs α in our complex (Supplemental Fig. 2). Furthermore, A_2A_AR-Gs α:β_4_γ_2_ is prenylated, whereas A_2A_AR-miniGs α:β_1_γ_2_ contains a mutated Gγ_2_ isoform to prevent prenylation. Lastly, A_2A_AR-miniGs α:β_1_γ_2_ differs from A_2A_AR-Gs α:β_4_γ_2_ by binding NECA instead of UK-432,097, which has higher receptor affinity. Notwithstanding these differences, profound functional differences have been observed in reconstitution ^9^ and expression systems ^10^ that contained identical A_2A_AR, agonist and G protein subunits, with the Gβ_1_ or Gβ_4_ isoform being the only variable (Fig. 1A). Consequently, structural differences would be expected to arise from A_2A_AR-Gs complexes differing only by the Gβ isoform. Scrutiny of the structures reveals that Gβ_4_ elicits a dramatic increase in the solvent accessible volume within the complex (Fig. 2). Significant structural correlates are an extension of the Gs α helix HN and a switch at proline 285 in receptor helix TM7 (Fig. 4A, B). The mechanism for the increased distance between the Gβ_4_ torus and the Gs α Ras domain is not clear; however, it appears to be enabled by an elastic region of the Gs α HN that contacts ICL2 (Fig. 5B). In the case of A_2A_AR-Gs α:β_4_γ_2_, it is likely that the structural relationship between Gβ_4_ and Gs α expands this elastic region of HN, employing HN as an “actuator switch” to enlarge the interior cavity of A_2A_AR via interactions with ICL2 in a process that is accommodated by the proline 285 switch (Fig. 4A, B).

The increased separation between Gs α and Gβ in A_2A_AR-Gs α:Gβ_4_γ_2_ versus A_2A_AR-miniGs α:Gβ_1_γ_2_ (Fig. 5 and depicted as cartoons in Fig. 6A), suggests a mechanism for the tenfold increased coupling efficiency of Gβ_4_γ_2_ over Gβ_1_γ_2_ in catalyzing A_2A_AR dependent GTPγS binding to Gs α ^9^ (Fig. 1A). In particular, the conformational change required of switch II to bind GTP would be less constrained by Gβ_4_ (Fig. 6B). In contrast, tighter interactions between miniGs α and Gβ_1_ (Fig. 6C) would provide a higher energy barrier for switch II to undergo the GTP dependent conformational change. Fig. 6D illustrates H3 and Switch II of the GDP bound Gs α:β_1_γ_2_ heterotrimer ^19^, highlighting the proximity of Switch II with Gβ_1_. In contrast, Fig. 6E shows the same orientation of the GSP activated Gs α subunit ^20^, with Switch II adopting a different conformation. Note that R231^G.H2.04^ of Switch II binds to Gβ_1_ in the GDP bound form of Gs α (Fig. 6D), but swings around to form a salt bridge with E268^G.H3.04^ of H3 in the activated GSP bound state (Fig. 6E); this ionic bond has been referred to as a “hasp,” and was determined to be necessary to stabilize the GTP bound form of Gs α ^21^. Thus, the nature of the Gs α:Gβ interface is likely related to the kinetics of nucleotide binding, with a more open interface allowing more rapid binding of GTP. Whether the effect of Gβ_4_ on the active states of GPCRs extends beyond A_2A_AR has yet to be determined. Intriguingly, reconstitution experiments have shown that the Go α:β_4_γ_2_ heterotrimer displayed an increased level of M2 Receptor (M2R) dependent nucleotide exchange compared to the Go α:β_1_γ_2_ heterotrimer, suggesting an enhanced ability of Gβ_4_ to activate M2R ^22^.

We propose that the enhancement of signaling efficiency by Gβ_4_ versus Gβ_1_ is a result of the increased solvent accessible volume in the complex with Gβ_4_ versus Gβ_1_. This observation that occurs on the cytoplasmic side of the A_2A_AR complex is remarkably parallel to the activity of full and partial agonists in the orthostatic site on the extracellular side of A_2A_AR. One possible explanation for the enhanced signaling efficiency of full vs partial agonists was proposed in a study that observed different conformations and levels of signaling efficiency of A_2A_AR, depending on the specific agonist ^8^. Modulation of active states may be similarly occurring on the cytoplasmic side of A_2A_AR. That is, the effect of Gβ_4_ is analogous to a full agonist, inducing an active state with higher signaling activity than Gβ_1_, which would be analogous to a partial agonist.

## Materials and Methods

### Design of the A_2A_AR and Gs constructs

The wild-type A_2A_AR construct for cryoEM contained a hemagluttinin (HA) signal sequence, a FLAG epitope, a TEV cleavage site and a T4 lysozyme sequence, which was attached to the amino terminus of A_2A_AR lacking the first four residues. The C-terminus was truncated at position 316, and the final construct was designated HA-Flag-TEV-T4L-A_2A_AR-316. The difference for the construct used for X-ray crystallography was that T4 lysozyme was inserted in the third intracellular loop rather than at the amino terminus ^14^. The Gs heterotrimer contained human Gs α, Gβ_4_ containing a V226E mutation in order to bind a stabilizing nanobody (Nb35) which was used for stabilizing the β_2_-adrenergic receptor Gs complex ^23^, and an amino-terminally 6His-FLAG tagged human Gγ_2_^24^. The plasmids containing Nb35 was kindly provided by S.G. Rassmussen (Stanford University). A Gγ_2_ clone from bovine brain^25^ (which translates to the same protein sequence as the human Gγ_2_) was ligated into the pDoubleTrouble vector (pDT)^26^ in order to add a 6His-FLAG tag to the N-terminus of Gγ_2_ (Gγ_2FH_)^24^. The Gγ_2FH_ coding sequence was subcloned into the transfer vector pVL1393, and recombinant baculoviruses were isolated as described ^24^.

### Insect cell expression and purification of A_2A_AR, the Gs heterotrimer and Nb35

A_2A_AR and the Gs heterotrimer were expressed separately in Sf9 (*S. frugiperda*) insect cells using the Bac-to-Bac Baculovirus Expression System (Invitrogen). For A_2A_AR, Sf9 cells were grown in ESF921 media (Expression Systems) at 27 °C, diluted to a density of 2.0 × 10^6^ cells/mL and infected for 48 hrs with a high-titre baculovirus stock at a multiplicity of infection (MOI) of 3, harvested by centrifugation and stored at −80 °C. Viral titers were performed by a flow cytometric method ^27^ on a Guava easycyte 8HT after staining the cells with anti-gp64-PE antibody (Expression Systems.) For the Gs heterotrimer, Sf9 cells were diluted to a density of 2.5 × 10^6^ cells/mL and then infected with three separate baculoviruses (Gs α, Gβ_4_ and Gγ_2FH_) as described ^9^.

Membranes were prepared from receptor-infected insect cells based on the method by Jaakola *et al.* ^28^. All steps were performed at 4 °C unless otherwise stated. Cells were resuspended in a hypotonic Buffer A (10 mM HEPES, pH 7.5, 10 mM MgCl_2_, 20 mM KCl and EDTA-free protease inhibitor cocktail (Roche)) and lysed by Dounce homogenization (~20 strokes/wash); membranes were recovered by centrifugation at 100,000 x g and washed again with Buffer A. Two additional washes were performed with buffer A containing 1M NaCl (Buffer B). After the final wash, membranes were weighed, resuspended in 2.5 volumes of Buffer A containing 40% (v/v) glycerol (Buffer C), and flash frozen in liquid nitrogen.

For purification of A_2A_AR, frozen membranes were thawed in a 10-fold excess of 20 mM HEPES, pH 7.5, 10% glycerol, 4 mM CaCl_2_, 100 μM adenosine and EDTA-free protease inhibitor cocktail (Roche) (Buffer D) containing 300 mM NaCl and extracted with 0.5% n-dodecyl-β-D-maltoside/cholesteryl hemisuccinate (DDM/CHS) for 4 hrs with gentle rotation. Receptor extracts were clarified by high-speed centrifugation at 100,000 x g and then added to a slurry of anti-FLAG M1 agarose affinity gel (Sigma-Aldrich, catalog number A4596) for overnight binding with gentle rocking at 4 °C The slurry was collected in a column at 1 g, and the beads with bound A_2A_AR were washed with 30 column volumes of Buffer D containing 300 mM NaCl and 0.1% DDM/CHS, followed by a wash with 30 column volumes of Buffer D containing 500 mM NaCl and 0.05% DDM/CHS. The final wash was with 20 column volumes of Buffer D containing 150 mM NaCl and 0.025% DDM/CHS. The FLAG-M1 affinity resin containing bound A_2A_AR was then combined with purified Gs to assemble the A_2A_AR-Gs complex (described below).

Sf9 cell pellets containing Gs α, β_4_ and an N-terminally 6HIS-FLAG tagged γ_2_ were lysed in a five-fold excess of buffer containing Buffer E (20 mM Tris, pH 8.0, 10 μM GDP and EDTA-free protease inhibitor cocktail). All steps were performed at 4 °C unless otherwise stated. Unbroken cells and debris were removed by centrifugation at 2,500 x g for 10 min, and membranes were collected from the supernatant by centrifugation at 100,000 x g. Membranes containing Gs were suspended and extracted with Buffer E containing 100 mM NaCl and 1% cholate ^9^ by gentle rotation for 1 hr. The cholate extract was clarified by centrifugation at 100,000 x g, supplemented with 20 mM imidazole, and loaded onto a Ni-NTA superflow (Qiagen) column equilibrated with Buffer E. Contaminants were removed by washing with 8 column volumes of Buffer F (20 mM HEPES, pH 7.5, 500 mM NaCl, 0.5% Genapol C-100 (Sigma-Aldrich), containing 1 mM MgCl_2_, 10 μM GDP, 20 mM imidazole and EDTA-free protease inhibitor cocktail. Detergent was exchanged by washing with 1.5 column volumes of Buffer G (20 mM HEPES, pH 7.5, 100 mM NaCl, 0.1% DDM/CHS, 1 mM MgCl_2_, 10 μM GDP, and EDTA-free protease inhibitor cocktail), and the heterotrimeric Gs was eluted with Buffer G containing 200 mM imidazole. Elution fractions containing protein were identified by SDS-PAGE and pooled and concentrated using an Amicon Ultra-15 50,000 MWCO (Millipore). Concentrated protein was diluted with Buffer H (20 mM HEPES, pH 7.5, 20 mM NaCl, 0.025% DDM/CHS, 10 mM MgCl_2_, 1 mM EDTA, 100 μM TCEP and EDTA-free protease inhibitor cocktail) to lower the salt concentration, and the solution was loaded onto a 1.3 mL UnoQ Q1 column (BioRad). Protein was eluted with a linear gradient of 0% Buffer H to 40% Buffer I (Buffer H with 1M NaCl instead of 20 mM NaCl) over 20 mL. Collection wells contained a 10x solution of GDP to yield a final concentration of 10 μM. Fractions containing Gs were pooled, concentrated using an Amicon Ultra-4 50,000 MWCO (Millipore), aliquoted, flash frozen in liquid nitrogen and stored at −80 °C. The 6His tagged nanobody Nb35 was expressed in *E.coli* and purified by Immobilized Metal Affinity Chromatography as described *29.*

### Purification of A_2A_AR-Gs complexes

Formation of A_2A_AR-Gs complex utilized the washed A_2A_AR bound to FLAG-M1 affinity resin (described above) and purified Gs at a ratio of 1:1.5 A_2A_AR:Gs α, based on Simply Blue staining of A_2A_AR and Gs α gel bands. Excess GDP was removed from purified Gs by applying protein to Dowex 1×4 ion exchange resin (Sigma-Aldrich, catalog number 428612) and eluting with Buffer J (25 mM HEPES, pH 7.5, 150 mM NaCl, 10% glycerol, 0.025% DDM/CHS, 1 mM MgCl_2_, 4 mM CaCl_2_, 100 μM adenosine and EDTA free protease inhibitor cocktail). Fractions containing Gs were pooled and combined with the A_2A_AR bound to FLAG-M1 affinity resin. Apyrase (7.5 units; NEB catalog number MO398L) and PNGaseF (500 units; NEB catalog number PO7O4S) were added to the reaction mixture, which was allowed to incubate at room temperature for 1 hr with gentle rotation. Nanobodies (~2-fold excess) were also added during this incubation.

The FLAG-M1 affinity resin containing A_2A_AR-Gs complex and nanobodies was then washed with 10 mL of Buffer J in a column at 1 g to remove contaminants, which included excess Gβγ, due to the location of the affinity tag on Gγ. A_2A_AR-Gs complex was eluted with Buffer J containing 10 mM EDTA; collection tubes contained MgCl_2_ to give a final concentration of 15 mM MgCl_2_. Eluted A_2A_AR-Gs complex was pooled, and 10 μM UK-432,097 (Axon Medchem) was added to displace bound adenosine. Protein was concentrated using a Vivaspin 6 100,000 MWCO (GE Healthcare), and success of complex formation was initially judged by SEC using a Superdex 200 10/300 GL column (GE Healthcare Life Sciences) equilibrated with Buffer K (25 mM HEPES, pH 7.5, 100 mM NaCl, 0.025% DDM/CHS, 10 mM MgCl_2_, 1 mM EDTA, 100 μM TCEP and 1 μM UK-432,097. Peak fractions of the SEC purified A_2A_AR-Gs complex were pooled and crosslinked with 0.1% glutaraldehyde (SigmaAldrich) for 30 min at 4 °C, quenched with 0.1 M Tris, pH 8.0, and concentrated to ~400 μl. The amphipol A8-35 was then added to the A_2A_AR-Gs complex (100 µl of 10 mg/mL stock prepared using Buffer K without DDM/CHS) and incubated overnight at 4 °C with gentle rotation. Detergent was removed by a final SEC purification using Buffer K without DDM/CHS. Peak fractions of the A_2A_AR-Gs complex were pooled and concentrated in a 0.5 mL Vivaspin 100,000 MWCO concentrator prior to application onto cryoEM grids.

### Radioligand binding assay

Antagonist competition assays were performed on ~50 μg of T4L-A_2A_AR-Gs α:β_4_γ_2_ complex purified by SEC in Buffer L (25 mM HEPES, pH 7.5, 100 mM NaCl, 0.1% DDM/0.02%CHS, 10 mM MgCl_2_, 1 mM EDTA, 100 μM TCEP, 100 μM adenosine and EDTA-free protease inhibitor cocktail). Adenosine deaminase (Roche, 2.5 units/mL) and ~2 µl [^3^H] ZM241385 (American Radiolabeled Chemicals, Inc.) were combined with Buffer L without adenosine and distributed evenly among reaction tubes (5 μl/tube). Serial dilutions of non-radiolabel(NECA) were then added to the reaction tubes (5 μl/tube). The purified T4L-A_2A_AR-Gs α:β_4_γ_2_ complex was diluted with Buffer L without adenosine in the presence and absence of 100 μM GTPγS (final concentration) and added to the reaction tubes (20 μl and ~1 μg/tube) using pipetting to mix. After a 3-hr incubation at room temperature, the reaction was terminated by vacuum filtration using 0.1 μM VCWP filters (Millipore), which were washed with ice-cold Buffer M (20 mM HEPES, pH 7.5, 100 mM NaCl and 10 mM MgCl_2_). The activity of [^3^H] ZM241385 binding was determined by liquid scintillation counting. Radioligand binding data were fitted to a two-site competition, least-squares fit using Graph Pad Prism 9.5.1.

### CryoEM grid preparation

CryoEM grids were prepared in the University of Virginia Molecular Electron Microscopy Core (MEMC) using a Vitrobot Mark IV with the blotting chamber at 4°C and 95 % relative humidity. Whatman #1 filter paper was used to blot grids using a blot force of 7 and blot time of 14 s. UltrAuFoil 1.2/1.3, 300 mesh grids (Quantifoil) were glow discharged in a Pelco EasiGlow for 60 s at 20 mA. A 2.1 µL aliquot of A_2A_AR-Gs was applied to the gold-film side of each grid and vitrified in liquid ethane cooled by liquid nitrogen.

### CryoEM data collection

CryoEM data were collected in the University of Virginia Molecular Electron Microscopy Core (MEMC) on a ThermoFisher Scientific Titan Krios equipped with a Gatan BioQuantum-K3 energy filter and detector (Supplemental Table 1).

### CryoEM image processing and reconstruction

All processing was performed in cryoSPARC 3.0.0 (DOI: 10.1038/nmeth.4169). Patch motion correction in cryoSPARC Live was performed during data collection to assess grid and sample quality. Following data collection, processing was done in the standard cryoSPARC suite. CTFFIND4 was used for CTF estimation (https://doi.org/10.1016/j.jsb.2015.08.008). 3,873 micrographs with defocus values between −2.5 and −0.8 µm and estimated maximum resolution better than 3.7 Å were used for further processing. Blob picking with 100 micrographs resulted in 54,657 particles that were extracted with a box size of 256 pixels (358.4 Å). 2D classification with these particles was used to generate five templates that were used for template-based particle picking from all 3,873 micrographs. Template particle picking followed by particle inspection with NCC=0.4 and local power between 121 and 155 resulted in 2,600,922 particles. Particle extraction from 1000 micrographs with a box size of 256 pixels followed by 2D classification and selection resulted in 355,217 particles that were used to generate three *ab initio* reconstructions. Particles were extracted from the remaining micrographs and ARG particles were purified by 40 iterations of 2D classification using 100 classes and a circular mask of 200 Å. 2D class selection resulted in 1,143,600 particles that were subjected to heterogeneous refinement using the three *ab initio* reconstructions. The best 3D class contained 545,805 particles that were used for non-uniform refinement that resulted in a map with a cryoSPARC GSFSC resolution of 3.4 Å (FSC=0.143). These particles were subjected to heterogeneous refinement with 6 classes and one class containing 123,274 particles. These particles were used for another round of non-uniform refinement that resulted in a map with a cryoSPARC GSFSC resolution of 3.5 Å (FSC=0.143) but that was qualitatively better in some regions. This map was used for local resolution estimation followed by local map filtering. The non-uniform refinement and the locally filtered Coulomb potential maps were used for model building and refinement. These Coulomb potential maps were also used to generate charge density maps in Chimera (DOI: 10.1016/j.sbi.2019.04.006, https://doi.org/10.1002/pro.3198, https://doi.org/10.1002/jcc.20084).

### Model building and refinement

We constructed a model of the A_2A_AR-Gs complex, using a combination of X-ray crystallography and homology modeling, at a time when the only experimentally determined GPCR-G protein structure was the X-ray structure of β_2_AR-Gs (**PDB:3SN6**)^23^. The agonist-bound A_2A_AR X-ray structure had also been solved (**PDB:3QAK**)^14^, but we also solved the X-ray structure of agonist-bound A_2A_AR in the course of our work on solving the A_2A_AR-Gs complex structure by X-ray crystallography. Briefly, we expressed, purified, and crystallized agonist-bound (UK-4320097) A_2A_AR-T4L-ΔC as previously described ^14^ with minor modifications. Purified A_2A_AR-T4L-ΔC-UK-432,097 in DDM/CHS was reconstituted into a 90%/10% monoolein/cholesterol mixture and Pn3m lipidic cubic phase (LCP) by mixing in two 100 uL coupled Hamilton syringes. Screening around the published crystallization condition yielded A_2A_AR-T4L-ΔC crystals, but larger, single crystals were grown using 40 % PEG 300, 0.5 M K formate, 0.1 M Na citrate pH 4.5. A_2A_AR-T4L-ΔC crystals were harvested from glass sandwich plates with Mitegen loops, frozen in liquid nitrogen, and sent to the SER-CAT APS synchrotron beamline for remote X-ray diffraction data collection. X-ray diffraction data from five A_2A_AR-T4L-ΔC crystals were indexed, integrated, and scaled in HKL-2000 (https://doi.org/10.1016/S0076-6879(97)76066-X) and merged in CCP4 (doi:10.1107/S0907444910045749, doi: 10.1107/S0907444905036693). Phases were determined by molecular replacement using the program Phaser in Phenix (DOI: 10.1107/S0021889807021206, doi: 10.1107/S2059798319011471) using the 3QAK model. The model was refined with Phenix refine (DOI: 10.1107/S0907444912001308) and real space refinement in COOT (doi: 10.1107/S0907444904019158). The refined A_2A_AR model was superposed with the β_2_AR-Gs model in COOT using SSM (doi: 10.1107/S0907444904026460). The cytoplasmic ends of A_2A_AR TM5/6 were deleted from the model where they deviated significantly from the β_2_AR-Gs model. A homology model of these portions of A_2A_AR TM5/6 was generated using the corresponding fragment of the β_2_AR-Gs model and the program Swiss-model (https://doi.org/10.1093/nar/gky427), and the active-state conformation of the A_2A_AR TM5/6 cytoplasmic ends were recombined with the A_2A_AR crystal structure. A homology model of Gs α:β_4_γ_2_ was generated using Swiss-model and the Gs α:β_4_γ_2_ structure from 3SN6^23^. The Gs α:β_4_γ_2_ homology model was combined with the hybrid X-ray/homology model of A_2A_AR in COOT to complete the initial A_2A_AR-Gs model. The A_2A_AR-Gs homology model was computationally reconstituted into a POPC bilayer, solvated, and ionized (140 mM NaCl) using CHARMM-GUI (https://doi.org/10.1002/jcc.20945) resulting a system comprised of ~330,000 atoms. UK-432097 was parameterized using CGenFF (DOI: 10.1002/jcc.21367). NAMD (doi:10.1063/5.0014475) was used with the CHARMM36 force field to equilibrate the system as an NPT ensemble followed by minimization and unrestrained all-atom simulation (40 ns) as an NVT ensemble with T=310 K. Nb35 was manually added to the MD-minimized A_2A_AR-Gs model based on the position of the nanobody in the β_2_AR-Gs structure. The A_2A_AR-Gs-Nb35 model was docked into the A_2A_AR-Gs cryoEM map using Phenix Dock In Map. The map also revealed densities consistent with cholesteryl-hemisuccinate (CHS) molecules. CHS molecules were extracted from **PDB:7D76** (DOI: 10.1038/s41586-020-03083-w) and manually docked into the Coulomb potential map, five at the extracellular ends of TM1-4, three at the cytoplasmic ends of these helices and two at the extracellular end of TM6 and ECL3. The A_2A_AR-Gs-Nb35 model was refined using alternating cycles of Phenix Real Space Refine and manual real space refinement in COOT.

### Surface area of solvent accessibility calculations

Prior to surface area calculations, missing side chains were added to the structures using the Protein Repair & Analysis Server www.protein-science.com. The solvent accessible surface areas (SASA) of the A_2A_AR-Gs α:β_4_γ_2_ and A_2A_AR-miniGs α:β_1_γ_2_ (**PDB:6GDG**) structures, were calculated on a per residue basis and expressed as a percent ratio. The calculations used the GETAREA server http://curie.utmb.edu/getarea.html, with 1.4 Å assigned as the radius of the probe water molecule. SASA values per residue from the A_2A_AR-Gs α:β_4_γ_2_ were divided by SASA values from the analogous residues in the A_2A_AR-miniGs α:β_1_γ_2_ structure to generate ratios that allowed comparison of the differences in SASA between the structures, Values were excluded from analysis if the ∆ between the structures was <2.5%.

## Supplementary Material

Supplement 1 **Supplemental Fig. 1 Purification and functional characterization of T4L-A**_**2A**_**AR-Gs α:β**_**4**_**γ**_**2**_ **complex used in structural studies. (A)** Workflow of T4L-A_2A_AR-Gs α:β_4_γ_2FH_ complex formation. (**B**) Simply Blue stained SDS-PAGE gel of purified T4L-A_2A_AR and Gs α:β_4_γ_2FH_. (**C**) SEC analysis of T4L-A_2A_AR-Gs α:β_4_γ_2FH_ complex; fractions separated by SDS-PAGE and stained with silver. (**D**) Simply Blue stained gel of SDS-PAGE separated T4L-A_2A_AR-Gs α:β_4_γ_2_ complex with nanobody Nb35 bound. (**E**) Displacement of antagonist [^3^H] ZM241385 binding to the T4L-A_2A_AR-Gs complex by agonist NECA in the absence of GTPγS; 66.1% of A_2A_AR was in the high affinity state (EC_50_ = 157.7 nM) and 33.9% of A_2A_AR was in the low affinity state (EC_50_ = 6.0 mM). In the presence of GTPγS, the NECA displacement curve shifts to the right; 19.8% of A_2A_AR was in the high affinity state (EC_50_ = 16.4 nM) and 80.2% of A_2A_AR was in the low affinity state (EC_50_ = 8.5 mM).
**Supplemental Fig. 2 Sequence alignment of full length Gs α with miniGs α.** The multiple sequence alignment program Clustal Omega was used to align the full-length short form of human Gs α used in this study with the thermostabilized miniGs α construct used in the A_2A_AR-miniGs α:β_1_γ_2_ structure^13^ (**PDB: 6GDG**).
Supplemental Table 1. CryoEM data and statistics.

## Data Availability

The atomic coordinates of A_2A_AR-T4L-ΔC with bound UK-432,097 have been deposited in the Protein Data Bank (www.rcsb.org) with accession code XXXXX. The coordinates for the A_2A_AR-Gs a:b_4_g_2_ complex have been deposited in EMDB (www.ebi.ac.uk/pdbe/emdb/) with accession code XXXXX.

## Significance Statement

Almost all GPCR complex structures to date contain a heterotrimeric G protein with the Gβ_1_ isoform. Compared with complexes with the Gβ_1_ isoform, the A_2A_AR-Gs α:β_4_γ_2_ complex displays a greater solvent accessible surface area that we propose accounts for the ten-fold increased efficiency of Gβ_4_γ_2_ over Gβ_1_γ_2_ in catalyzing A_2A_AR dependent GTPγS binding to Gs α.
