# Supplementary material for: G protein β4 as a structural determinant of enhanced nucleotide exchange in the A2AAR-Gs complex
Source: Res Sq. 2024 Jan 23:rs.3.rs-3814988. Preprint. [Version 1] doi: 10.21203/rs.3.rs-3814988/v1 (PMC10854301; doi:10.21203/rs.3.rs-3814988/v1)
Supplement: Supplement 1 — Supplemental Fig. 1 Purification and functional characterization of T4L-A2AAR-Gs α:β4γ2 complex used in structural studies. (A) Workflow of T4L-A2AAR-Gs α:β4γ2FH complex formation. (B) Simply Blue stained SDS-PAGE gel of purified T4L-A2AAR and Gs α:β4γ2FH. (C) SEC analysis of T4L-A2AAR-Gs α:β4γ2FH complex; fractions separated by SDS-PAGE and stained with silver. (D) Simply Blue stained gel of SDS-PAGE separated T4L-A2AAR-Gs α:β4γ2 complex with nanobody Nb35 bound. (E) Displacement of antagonist [3H] ZM241385 binding to the T4L-A2AAR-Gs complex by agonist NECA in the absence of GTPγS; 66.1% of A2AAR was in the high affinity state (EC50 = 157.7 nM) and 33.9% of A2AAR was in the low affinity state (EC50 = 6.0 mM). In the presence of GTPγS, the NECA displacement curve shifts to the right; 19.8% of A2AAR was in the high affinity state (EC50 = 16.4 nM) and 80.2% of A2AAR was in the low affinity state (EC50 = 8.5 mM). Supplemental Fig. 2 Sequence alignment of full length Gs α with miniGs α. The multiple sequence alignment program Clustal Omega was used to align the full-length short form of human Gs α used in this study with the thermostabilized miniGs α construct used in the A2AAR-miniGs α:β1γ2 structure13 (PDB: 6GDG). Supplemental Table 1. CryoEM data and statistics. [file NIHPPrs3814988v1-supplement-1.pdf]

G protein β_4 as a structural determinant of enhanced nucleotide exchange in the A_{2A}AR-Gs complex
Supplementary Data

William E. McIntire[1†], Michael D. Purdy[2,3†], Susan A. Leonhardt[1†], Iga Kucharska[1], Michael A. Hanson[1], Sandra Poulos[2], James C. Garrison[4], Joel Linden[2] and Mark Yeager[1]

[1]The Phillip and Patricia Frost Institute for Chemistry and Molecular Science, University of Miami, Coral Gables, Florida 33146.

[2]Department of Molecular Physiology and Biological Physics, University of Virginia School of Medicine, Charlottesville, VA 22908 USA.

[3]Molecular Electron Microscopy Core, University of Virginia School of Medicine, Charlottesville, Virginia 22908, USA.

[4]Department of Pharmacology, University of Virginia Health System, Charlottesville, VA 22 903
Virginia 22908, USA.

† These authors contributed equally to this work.

*Correspondence and requests for materials should be addressed to M.Y.

Mark Yeager, M.D., Ph.D.
The Phillip and Patricia Frost Institute for Chemistry and Molecular Science
1201 Memorial Drive
University of Miami
Miami, FL 33146

Phone: 858-344-1834
E-mail: yeager@miami.edu

THE PHILLIP and PATRICIA FROST
INSTITUTE for CHEMISTRY
and MOLECULAR SCIENCE

UNIVERSITY
OF MIAMI

Supplemental Figure 1

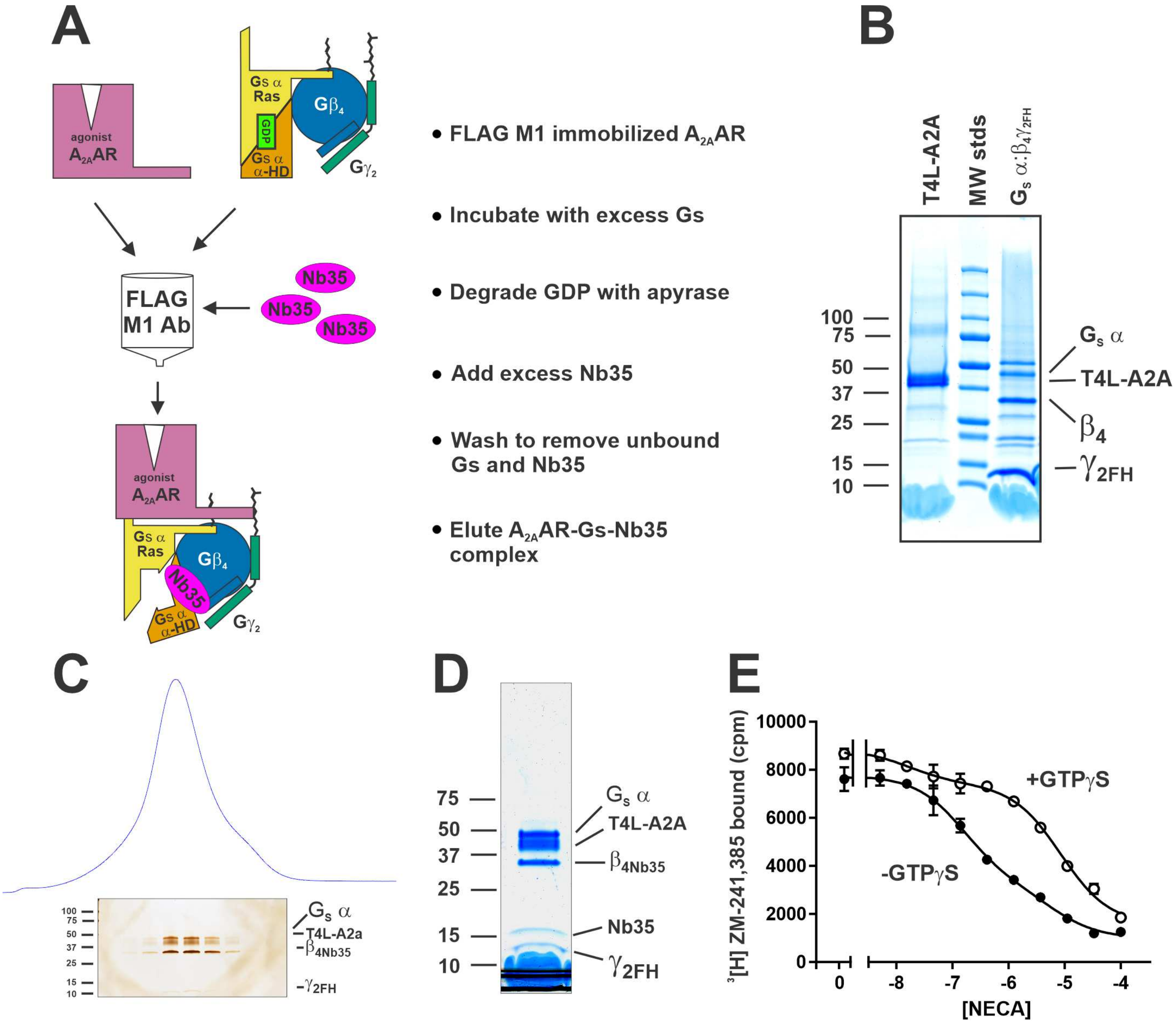

Supplemental Fig. 1 | Purification and functional characterization of T4L-A_{2A}AR-Gs α:G$\beta_4\gamma_2$ complex used in structural studies. (**A**) Workflow of T4L-A_{2A}AR-Gs α:$\beta_4\gamma_{2FH}$ complex formation. (**B**) Simply Blue stained SDS-PAGE gel of purified T4L-A_{2A}AR and Gs α:G$\beta_4\gamma_{2FH}$. **(C)** SEC analysis of T4L-A_{2A}AR-Gs α:$\beta_4\gamma_{2FH}$ complex; fractions separated by SDS-PAGE and stained with silver. **(D)** Simply Blue stained gel of SDS-PAGE separated T4L-A_{2A}AR- Gs α:$\beta_4\gamma_2$ complex with nanobody Nb35 bound. **(E)** Displacement of antagonist [^{3}H] ZM241385 binding to the T4L-A_{2A}AR-Gs complex by agonist NECA in the absence of GTPγS; 66.1% of A_{2A}AR were in the high affinity state (EC_{50} = 157.7 nM) and 33.9% of A_{2A}AR were in the low affinity state (EC_{50} = 6.0 μM). In the presence of GTPγS, the NECA displacement curve shifts to the right; 19.8% of A_{2A}AR were in the high affinity state (EC_{50} = 16.4 nM) and 80.2% of A_{2A}AR were in the low affinity state (EC_{50} = 8.5 μM).

Supplemental Figure 2

```
Full length native Gs α   MGCLGNSKTEDQRNEEKAQREANKKIEKQLQKDKQVYRATHRLLLLGAGESGKSTIVKQM     60
miniGs α (PDB: 6GDG)      -----NSKTEDQRNEEKAQREANKKIEKQLQKDKQVYRATHRLLLLGADNSGKSTIVKQM     55
                               *******************************************.:**********

Full length native Gs α   RILHVNGFNGEGGEEDPQAARSNSDGEKATKVQDIKNNLKEAIETIVAAMSNLVPPVELA     120
miniGs α (PDB: 6GDG)      RILHGGS-GGSG------------------------------------------------     66
                          **** .. .*.*

Full length native Gs α   NPENQFRVDYILSVMNVPDFDFPPEFYEHAKALWEDEGVRACYERSNEYQLIDCAQYFLD     180
miniGs α (PDB: 6GDG)      ------------------------------------------------------------     66

Full length native Gs α   KIDVIKQADYVPSDQDLLRCRVLTSGIFETKFQVDKVNFHMFDVGGQRDERRKWIQCFND     240
miniGs α (PDB: 6GDG)      ----------------------GTSGIFETKFQVDKVNFHMFDVGGQRDERRKWIQCFND     104
                                                 *************************************

Full length native Gs α   VTAIIFVVASSSYNMVIREDNQTNRLQEALNLFKSIWNNRWLRTISVILFLNKQDLLAEK     300
miniGs α (PDB: 6GDG)      VTAIIFVVDSSDY----------NRLQEALNLFKSIWNNRWLRTISVILFLNKQDLLAEK     154
                          ******** **.*          *************************************

Full length native Gs α   VLAGKSKIEDYFPEFARYTTPEDATPEPGEDPRVTRAKYFIRDEFLRISTASGDGRHYCY     360
miniGs α (PDB: 6GDG)      VLAGKSKIEDYFPEFARYTTPEDATPEPGEDPRVTRAKYFIRDEFLRISTASGDGRHYCY     214
                          ************************************************************

Full length native Gs α   PHFTCAVDTENIRRVFNDCRDIIQRMHLRQYELL  394
miniGs α (PDB: 6GDG)      PHFTCAVDTENARRIFNDCRDIIQRMHLRQYELL  248
                          *********** **:*******************
```

Supplemental Fig. 2 Sequence alignment of full length Gs α with miniGs α. The multiple sequence alignment program Clustal Omega was used to align the full-length short form of human Gs α used in this study with the thermostabilized miniGs α construct used in the A_{2A}AR-miniGs α:$\beta_1\gamma_2$ structure[13] (**PDB: 6GDG**).

.

Supplemental Table 1

**Data Collection and Processing**	
Detector	Gatan K3
Energy Filter (Slit Width)	Gatan BioQuantum (10 eV)
Nominal Magnification	64000
Voltage (kV)	300
Electron exposure (e^{-}/A^2)	53
Defocus range (µm)	-2.2 to -1.0
Pixel size (Å)	1.4
Symmetry	C1
Total micrographs	6454
Selected micrographs	3873
Initial particles	2371061
Final particles	217489
Map resolution (Å)	3.5
FSC threshold	0.143
**Refinement**	
Initial model	In-house hybrid model*
Model resolution (Å)	3.5
FSC threshold	0.5
Map sharpening B factor ($Å^2$)	Local sharpening
Model composition	
Non-hydrogen atoms	8365
Protein residues	1057
Ligands	9
B factors ($Å^2$)	
Protein	42.7
Ligands	71.0
RMS deviations	
Bond lengths (Å) (# > 4σ)	0.002 (0)
Bond angles (Å) (# > 4σ)	0.580 (4)
Validation	
Molprobity score	1.75
Clashscore	7.77
Poor rotamers (%)	0
Ramachandran plot	
Favored (%)	95.4
Allowed (%)	4.5
Disallowed (%)	0.1
*see Methods section	

Supplemental Table 1 | CryoEM data and statistics.

Supplementary Files

This is a list of supplementary files associated with this preprint. Click to download.

- RS.pdf
- D9100089198valreportfullP1.pdf
- structuralfiles.zip
